# Transcriptomics profiling of Indian mustard (*Brassica juncea*) under arsenate stress identifies key candidate genes and regulatory pathways

**DOI:** 10.3389/fpls.2015.00646

**Published:** 2015-08-19

**Authors:** Sudhakar Srivastava, Ashish K. Srivastava, Gaurav Sablok, Tejaswini U. Deshpande, Penna Suprasanna

**Affiliations:** ^1^Nuclear Agriculture and Biotechnology Division, Bhabha Atomic Research CentreMumbai, India; ^2^Plant Functional Biology and Climate Change Cluster (C3), University of Technology SydneySydney, NSW, Australia; ^3^Shri Jagdishprasad Jhabarmal Tibrewala UniversityJhunjhunu, India

**Keywords:** arsenic, *Brassica juncea*, microarray, phytohormones, transporters, transposons

## Abstract

Arsenic (As) is a non-essential element, a groundwater pollutant, whose uptake by plants produces toxic effects. The use of As-contaminated groundwater for irrigation can affect the crop productivity. Realizing the importance of the *Brassica juncea* as a crop plant in terms of oil-yield, there is a need to unravel mechanistic details of response to As stress and identify key functional genes and pathways. In this research, we studied time-dependent (4–96 h) transcriptome changes in roots and shoots of *B. juncea* under arsenate [As(V)] stress using Agilent platform. Among the whole transcriptome profiled genes, a total of 1,285 genes showed significant change in expression pattern upon As(V) exposure. The differentially expressed genes were categorized to various signaling pathways including hormones (jasmonate, abscisic acid, auxin, and ethylene) and kinases. Significant effects were also noticed on genes related to sulfur, nitrogen, CHO, and lipid metabolisms along with photosynthesis. Biochemical assays were conducted using specific inhibitors of glutathione and jasmonate biosynthesis, and kinases. The inhibitor studies revealed interconnection among sulfur metabolism, jasmonate, and kinase signaling pathways. In addition, various transposons also constituted a part of the altered transcriptome. Lastly, we profiled a set of key functional up- and down-regulated genes using real-time RT-PCR, which could act as an early indicators of the As stress.

## Introduction

Crop productivity depends on factors like aeration, irrigation, and host–pathogen interactions, and also on presence/absence of abiotic and biotic stresses. Arsenic (As) is a highly toxic metalloid whose contamination is spread in large area of West Bengal, India, and Bangladesh. Such a widespread presence of As affects growth and yield of commonly cultivated crop plants in the area (Chaurasia et al., 2012; Kumar et al., 2015). In presence of As stress, an array of metabolic processes are affected and signs of As toxicity are often morphologically visible in terms of changes in root and shoot growth. Plants adapt several mechanisms of regulating the As tolerance viz., through altered expression of specific transporters or by modulating the epistatic interaction of the interconnected genes in pathways (Yu et al., 2012). Important steps of As tolerance have been identified as reduction of arsenate [As(V)] to arsenite [As(III)], organ- and tissue-specific and subcellular distribution of As, complexation with sulfur-containing ligands and vacuolar sequestration (Srivastava et al., 2012; Kumar et al., 2015). A fine coordination of these mechanisms to avoid As toxicity is achieved through transcriptome and proteome changes (Ahsan et al., 2008; Norton et al., 2008; Chakrabarty et al., 2009; Yu et al., 2012). However, plants suffer from toxicity when As accumulation surpasses a threshold level and particularly when its speciation dynamics are altered (Mishra et al., 2013).

An important step in As tolerance has been identified as “early sensing of the stress”. Srivastava et al. (2009) compared responses of tolerant and sensitive varieties of *Brassica juncea* and suggested early perception of As stress to be the cause of variable stress tolerance among different varieties. They suggested a hypothesis that the perception of As stress could be mediated by various hormones, which may sense As indirectly through its impact on sulfur metabolism. Other studies suggest that As(V) acts as a phosphate mimic and misleads metabolic and regulatory perception of itself as an abundant supply of phosphate and thus represses genes normally induced under low phosphate conditions (Catarecha et al., 2007; Abercrombie et al., 2008). In lieu of the above studies, it can be concluded that plants avoid extreme As toxicity since repression of phosphate uptake systems leads to reduced As(V) uptake as well (Catarecha et al., 2007). Castrillo et al. (2013) found that As(V) stress induces a notable transposon burst in plants, in coordination with As(V)/phosphate transporter repression, which immediately restricts As(V) uptake. They identified WRKY6 as an As(V)-responsive transcription factor that mediates As(V)/phosphate transporter gene expression and restricts As(V)-induced transposon activation. Other microarray and transcriptomic analyses in rice under As stress (Chakrabarty et al., 2009; Yu et al., 2012) implicated the role of various signaling molecules like abscisic acid (ABA), ethylene, cytokinins, salicylic acid (SA), flavonoids, and gibberellic acid (GA) in As stress responses of plants. In addition, various transcription factors, and protein kinases were found to be up- and down-regulated in response to As(V) and As(III).

*Brassica juncea* belonging to the family Brassicaeae represents one of the major oil-yielding crops in India and contributes 28.6% in the total oilseeds production and ranks second after groundnut sharing 27.8% in the India’s oilseed economy (Shekhawat et al., 2012). Srivastava et al. (2009) indicated an involvement of jasmonates in the signaling of As in *B. juncea.* Previous studies, conducted by our group on microRNA-specific microarray analysis of *B. juncea*, identified role of As-specific microRNAs in regulating sulfur metabolism, and metabolism and function of hormones like jasmonates, auxins, and ABA (Srivastava et al., 2013a). Taking into account all the studies, we understand that there is a need to reveal key candidate genes and pathways in *B. juncea* responsive to As stress that can also act as early As stress responsive markers in further studies. To identify such functional screening markers in root and shoot and to further enhance our understanding of As stress responses in *B. juncea*, we performed time-dependent transcriptome analysis of roots and shoots of *B. juncea* to understand the dynamic regulation of pathways involved in perception of and response to As stress and propose set of key genes and pathways.

## Materials and Methods

### Plant Material, As Treatment, and RNA Preparation

To study the response of the As stress, *B. juncea* (L.) Czern. var. TPM-1 was used as the plant material, which is an As tolerant variety. Seeds were sterilized and grown in a Plant Growth Chamber (Sanyo, Japan) as detailed previously (Srivastava et al., 2013a) having a daily cycle of a 14-h photoperiod with a light intensity of 150 μE m^-2^s^-1^, day/night temperature of 25 ± 2°C, and relative humidity of 65–75% for a week. After 12 days, seedlings were exposed to 500 μM arsenate [As(V); as Na_2_HAsO_4_] for 96 h. Seedlings were harvested for conducting microarray analysis at 4, 24, and 96 h and roots and shoots were separated and were used for RNA preparation. The quantity and purity of the RNA was determined by evaluating the absorbance at 260 nm and 260/280 nm absorbance ratio, respectively. Each of the total RNA preparations was individually assessed for RNA quality based on the 28S/18S ratio and RIN measured on an Agilent 2100 Bioanalyzer system using the RNA 6000 Nano LabChip Kit. With the use of Agilent’s 1-Color Quick Amp Labeling Kit, 500 ng of high quality total RNA was denatured in the presence of a T7 promoter primer and a 1-Color RNA Spike-In Kit. Reverse transcriptase was used to retrotranscribe the mRNA. cDNA was used as a template for *in vitro* transcription where a T7 RNA polymerase simultaneously amplified target material and incorporated cyanine 3-labeled CTP. Labeled cRNA was purified using spin columns from the Qiagen RNeasy Mini Kit and the quantity and quality of the cRNA was determined by Nanodrop ND-1000 UV–VIS spectrophotometer.

### Microarray Probe Design and Hybridization

For the design of the microarray probes, a total set of 53,939 sequences, which include expressed sequence tags (ESTs) and transcript sequences (mRNA) of *Brassica* sp., were downloaded from GenBank and clustered into unigenes using CAP3 (Huang and Madan, 1999). To avoid the formation of spurious assembly, the threshold value for the –*p* parameter, which represents the “overlap percent identity cutoff”, was fixed to 97. For the probeset construction, a total of 26,881 non-redundant unigenes obtained after clustering were used, which includes 1,720 *B. juncea*, 15,259 *Brassica Napus*, and 5,075 *Brassica rapa* sequences; and 6,456 from other *Brassica* species using the Agilent eArray best probe composition algorithm with the option to design multiple probes for each sequence. The probes obtained were printed using Agilent’s 4x44 array and a set of positive and negative controls were also added on to the microarray chip. To reduce the noise and the microarray hybridization bias, a set of replicate probes were also added for calculating the intra-array reproducibility.

For fragmentation, Agilent’s Gene Expression Hybridization Kit was used. Briefly, 1.65 μg of cyanine 3-labeled linearly amplified cRNA was added to hybridization cocktail and was then fragmented as per the manual of the Hybridization kit. The hybridization cocktail was then susbsequently dispensed into the wells of gasket slides and Brassica 4x44K Gene Expression Microarray was placed on the top of the gasket to allow for the hybridization. This microarray “sandwich” was then sealed in a hybridization chamber and was allowed to hybridize at 65°C by rotating at 10 RPM for 17 h. Following hybridization, slides were subsequently washed in Agilent’s Gene Expression Wash Buffers according to manufacturer specifications. Hybridized microarrays were scanned using the Agilent Microarray Scanner and spot intensities were analyzed using the Agilent Technologies Feature Extraction software version 10.7.3.1. Microarray data quality was evaluated by reviewing ten standard QC metrics generated by the Feature Extraction Software (Consult the Agilent Technologies Feature Extraction Software version 10.7.3.1 reference guide for a full explanation of the QC metrics). To rule out the possibilities of having a large hybridization, staining, or wash artifacts, array images were loaded into Feature Extraction Software for manual inspections. Microarrays were determined to be free of any large artifacts (≥10% of total surface area) that could have affected the quality of the data.

### Microarray Data Analysis

The spot intensity values obtained from Agilent Feature Extraction software were background corrected, and were normalized using Quantile Normalization. Principal Component Analysis and Correlation Analysis were used to identify outlier samples and to check the correlations between the samples. Statistical comparisons using a fold change ≥2 were carried out to identify the regulation of the genes under the As stress and to identify the candidate genes. Probes that were found to be significantly up- or down-regulated using the threshold mentioned above in each comparison were classified as putative markers of As stress. For functional assignments, the differentially expressed (DE) genes were BLASTed against *Arabidopsis thaliana* TAIR 10^1^ with an *E*-value of 1E-5, and in case of multiple hits, the corresponding hits were filtered based on e-values and % identity (See Supplementary **Data Sheet S1**). The genes showing up- and down-regulated profiles under the As treatment in root and shoot were assessed for the GeneSet Enrichment using assigned TAIR ids and PlantGSEA with cutoff value of 0.05 (Yi et al., 2013) Additionally, short time-series miner (STEM) analysis was done to cluster genes into specific profiles (Ernst and Bar-Joseph, 2006; Supplementary **Data Sheet S2**). The microarray data has been submitted to NCBI GEO (GSE66464). Further to develop network of interacting genes, methodology developed by Opgen-Rhein and Strimmer (2007) was used (Supplementary **Data Sheet S3**).

### Validation of Key Candidate Targets for Developing As Stress Early Indicators

All the primers used for SyBr green real-time RT-PCR were obtained from the *A. thaliana* RT-PCR primer pair database. The details of the primers used are mentioned in Supplementary **Table S1**. Specificity of all the primers was confirmed by sequence analysis of RT-PCR amplicons derived from *B. juncea* as detailed earlier (Srivastava et al., 2009). The DNA free total RNA was isolated from root samples (100 mg) and then quantitative real-time PCR was performed as described previously using a Corbett rotor gene 6000 (Corbett Life Science; www.corbettlifescience.com). The PCR cycling conditions comprised of 94°C for 5 min and 40 cycles each comprising of 94°C for 30 s, 55°C for 30 s, and 72°C for 30 s and final extension at 72°C for 10 min. For each sample, reactions were set up in triplicate to ensure the reproducibility of the results. Melting curves were generated and were analyzed using the dissociation curve software built into the Corbett rotor gene 6000. A relative expression ratio plot was generated using the software REST-MCS.

### Inhibitor Treatment and Biochemical and Transcriptional Assays

To ascertain the role of signaling mediated by jasmonate and kinases, and their interconnections with glutathione (GSH) metabolism, several inhibitors were employed. These included L-Buthionine Sulfoximine (BSO: a potent inhibitor of γ-glutamylcysteine synthetase, a rate-limiting enzyme of GSH biosynthesis), Ibuprofen (IBP: an inhibitor of lipoxygenase and hence the jasmonate biosynthesis), and Staurosporine (STS: an inhibitor of phospholipid/calcium-dependent protein kinases). Twelve days old seedlings, grown in hydroponics, as mentioned above, were used for the study. Treatment conditions included 500 μM As(V) plus a inhibitor viz., BSO (1 mM), IBP (25 μM), and STS (200 nM). For each treatment, a separate control was also maintained. Seedlings were pretreated with inhibitors for 16 h and then subjected to As(V) treatment for 24 h. The shoot samples of seedlings subjected to each of the treatments were harvested and were then used for various assays. The biochemical analyses included activity assay of enzymes and level of metabolites of sulfur metabolism. The activities of cysteine synthase and γ-glutamylcysteine synthetase were assayed according to Seelig and Meister (1984) and Saito et al. (1994), respectively, and the levels of cysteine and GSH were estimated according to the protocol of Gaitonde (1967) and Hissin and Hilf (1976), respectively, as detailed previously (Srivastava et al., 2009). The expression analysis of MAPK-3 and 12-oxophytodienoate reductase 1 (OPR-1) genes was done using real-time RT-PCR as per the protocol mentioned above and primer details are summarized in Supplementary **Table S1**.

## Results and Discussion

### Overall Pattern of Up- and Down-regulated Genes in Root and Shoot

**FIGURE 1:**
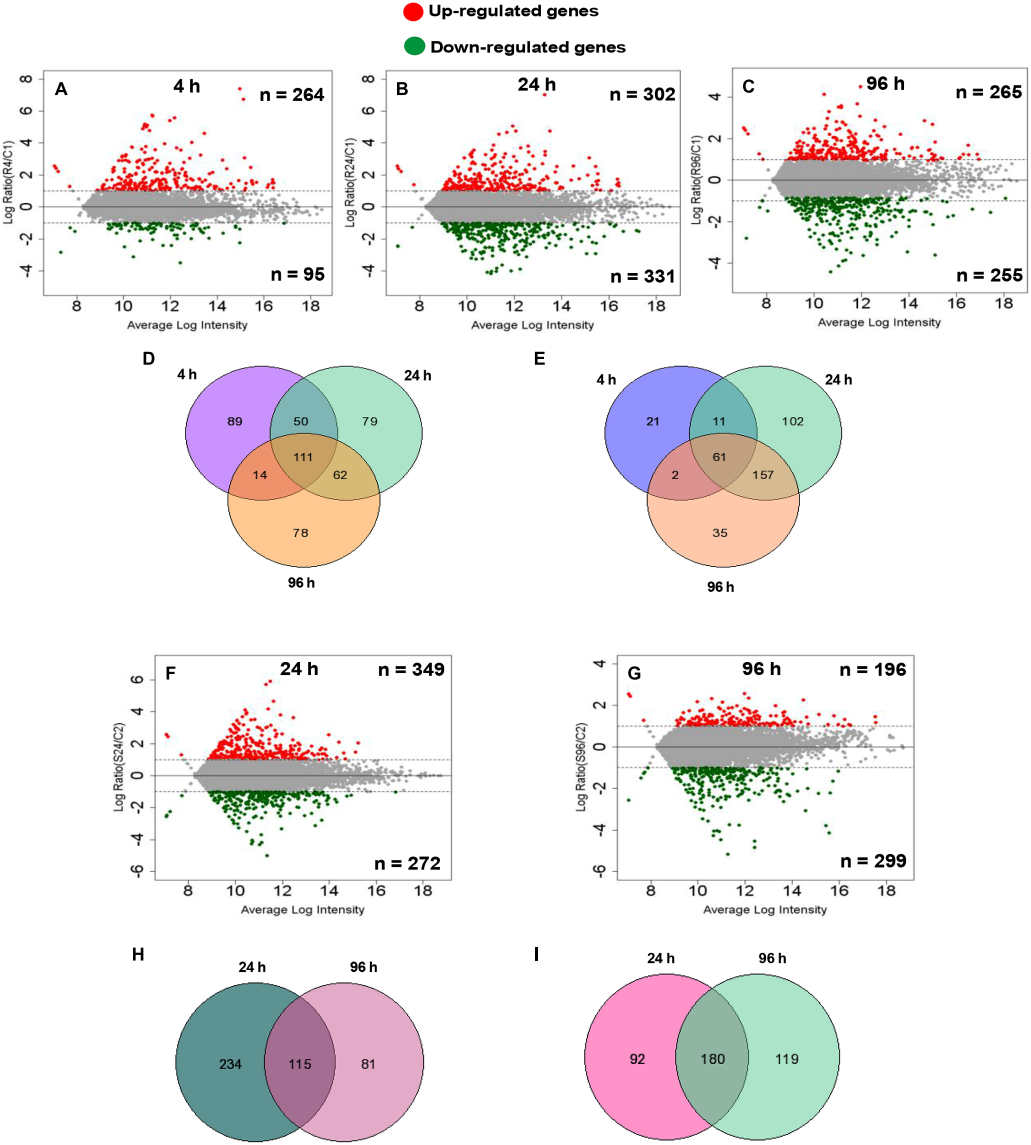
**Differentially expressed (DE) genes of root (*n* = 3) and shoot (*n* = 2) dataset at different time points compared with the control root and shoot samples calculated by difference **(A–C,F,G)** in arsenic-exposed *Brassica juncea* seedlings.** Scatter plot represents average intensity (*x*-axis) vs. log2 ratio (*y*-axis). Red and green colors define the up-regulated and down-regulated genes at each time point. Venn analysis of common and exclusively differential (up-regulated and down-regulated) genes in arsenic-exposed *B. Juncea* root (*n* = 3) **(D,E)** and shoot (*n* = 2) **(H,I)** samples.

To identify the important candidate genes, microarray profiling was done at 24 and 96 h for both roots and shoot. In addition, for roots, 4-h time point was also chosen so as to capture early responsive genes with a special reference to signaling related genes considering the fact that roots acts as the first contact point to As. At 4 h, a total of 359 genes showed significant change in expression with an up-regulation of 264 genes and down-regulation of 95 genes (**Figure 1A**). At 24 and 96 h, roots showed up-regulation of 302 and 265 genes, respectively, while down-regulation of 331 and 255 genes, respectively (**Figures 1B,C**). In shoots also, a large number of genes showed altered expression. A total of 349 and 196 genes were up-regulated and 272 and 299 genes were down-regulated at 24 and 96 h, respectively (**Figures 1F,G**). **Figures 1D,E,H,I** display the Venn diagram showing the amount of the co-expressed genes at each time point. Specifically, in roots, 111 genes were found to be up-regulated on all time points while 61 were found to be down-regulated. In shoots, commonly expressed up- and down-regulated genes were 115 and 180, respectively.

To profile the genes into respective clusters, microarray data was clustered using STEM tool (Ernst and Bar-Joseph, 2006). In roots, STEM analysis clustered the genes into 20 clusters from which six clusters were identified as significantly ordered based on their *p*-values. Among these clusters, significantly down-regulated genes were clustered into three profiles as, P0, P4, and P10, while up-regulated genes were clustered into P17, P18, and P19 (Supplementary **Figure S1**; Supplementary **Data Sheet S2**). These clusters include either down-regulated or up-regulated genes showing similar change in their expression and include genes of all studied time points. In shoot, regulated genes were profiled into 20 clusters from which 5 clusters were identified as significant ordered based on their *p*-values. Significantly down-regulated genes were clustered into P0 and P4, while up-regulated genes were clustered into P11, P14, and P15 (Supplementary **Figure S2**; Supplementary **Data Sheet S2**). The profile gene datasets were further analyzed to obtain gene–gene network interactions on their functional co-regulation pattern. In roots, down-(*n* = 312) and up-regulated (*n* = 314) profile gene datasets were analyzed that illustrated highly co-regulated 152 down-regulated (Supplementary **Figure S3**; Supplementary **Data Sheet S3**) and 145 up-regulated (Supplementary **Data Sheet S3**; Supplementary **Data Sheet S3**) genes (Opgen-Rhein and Strimmer, 2007). On the same front, down- (216) and up-regulated (255) profile gene datasets of shoots revealed highly co-regulated 162 down-regulated and 99 up-regulated genes (Supplementary **Figures S5** and **S6**; Supplementary **Data Sheet S3**). The data of co-regulated genes depicts about biological, cellular, and molecular processes regulated in a coordinated manner.

### Validation of the Set of Target Genes as Early Indicators

**FIGURE 2:**
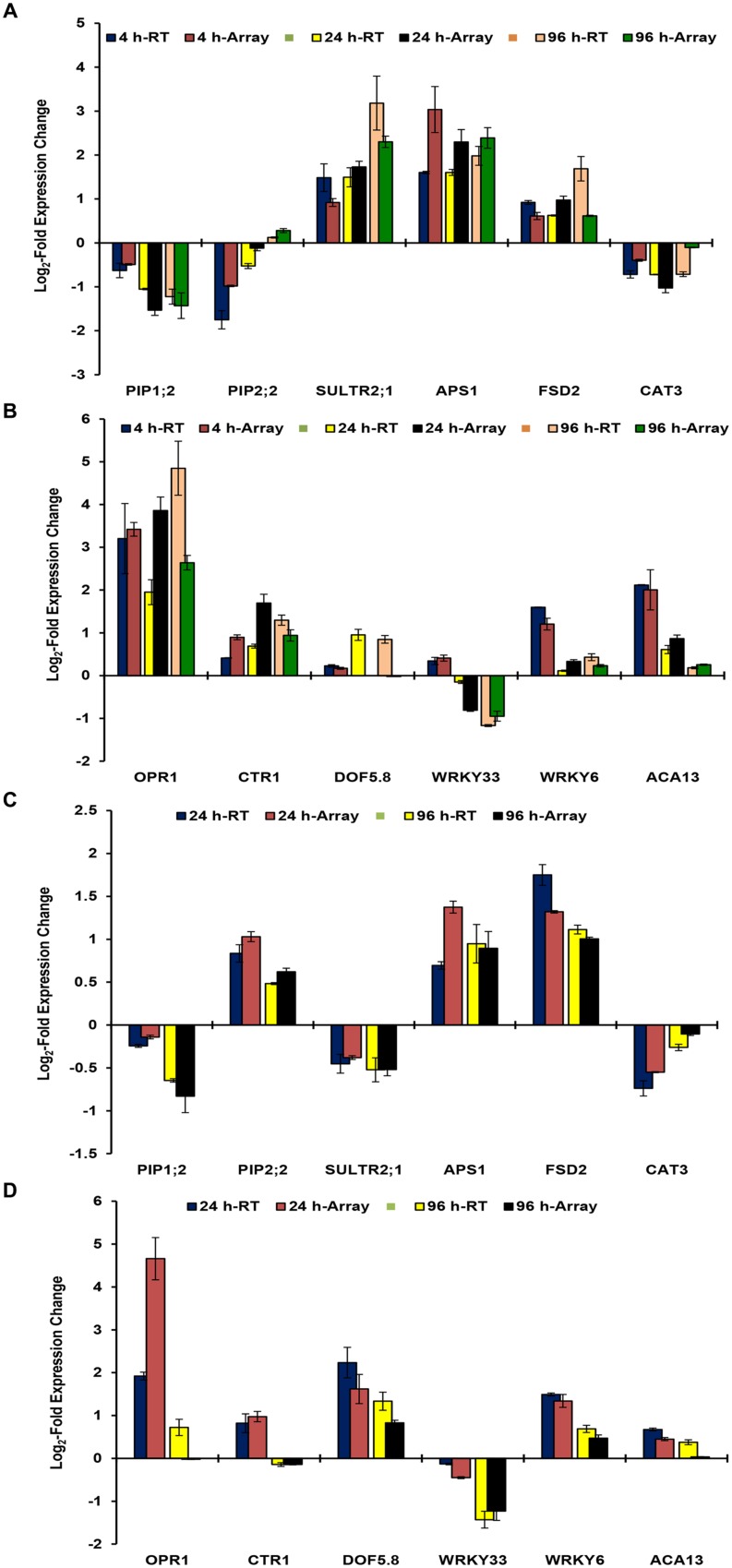
**Real time RT-PCR validation of microarray data of *B. juncea* roots at 4, 24, and 96 h (A,B); and of shoot at 24 and 96 h (C,D).** The data of RT-PCR has been overlapped with that microarray to demonstrate expression patterns. The *x*-axis represents the expression of various genes in control conditions.

Microarray data was validated by analyzing the expression patterns of selected genes in roots and shoots (**Figures 2A–D**). These genes have known important functions in As stress responses ranging from water homeostasis (PIP1;2 and PIP2;2; Srivastava et al., 2013b), sulfur transport and assimilation (SULTR2;1 and APS1; Srivastava et al., 2014), jasmonate signaling (OPR1; Srivastava et al., 2009), Ca signaling (ACA13; Rai et al., 2012), As(V) uptake and response (WRKY6; Castrillo et al., 2013), and antioxidant responses (FSD2 and CAT3) and signaling (CTR1 and WRKY33). The expression patterns of selected genes were found to be similar in real time RT-PCR analysis and microarray analysis for all time points. However, fold change levels were a bit different in some cases, which may be due to differences in sensitivity of different instruments, fluorescent dyes, and methods. Correlation analysis between real time RT PCR data and microarray data depicted significant positive correlation at *p*< 0.01 for 4 h (0.919), 24 h (0.911), and 96 h (0.922) for roots; and for 24 h (0.796) and 96 (0.944) for shoot.

### Gene Ontologies (GOs) and Enrichment Analysis of DE Genes

Transcriptomics offers an elusive way to look at the ubiquitous expression of genes and pathways specific to a particular stress conditions. To identify the genes, representative of particular pathways, SuperViewer^2^ was used and genes were classified on the basis of Mapman data. In addition, the up- and down-regulated genes were BLASTed against the TAIR 10 database and the corresponding *A. thaliana* IDs were used for gene-set enrichment using PlantGSEA. A total of 35, 4, 158, and 9 enriched gene sets in biological processes were found using *A. thaliana* as a background with an FDR < 0.05 in root down-regulated, root up-regulated, shoot down-regulated and shoot up-regulated genes, respectively (Supplementary **Data Sheet S3**). In root up-regulated geneset, enrichment analysis identified a GO category (GO:0009605, *p*-value 1.22E-04, FDR = 0.0338), which is linked to the salicyclic acid-mediated signaling pathway suggesting the up-regulation of the genesets involved in salicyclic acid singling. This observation of enriched GO of SA signaling correlates with the recent finding, where SA supplementation has been demonstrated to reduce the As toxicity by reducing the root to shoot translocation of As in *Oryza sativa* (Singh et al., 2015). Interestingly, less enriched genesets were observed in root down-regulated genes, which included GO:0034285 (Response to disaccharide stimulus, *p*-value = 1.61E-05, FDR = 0.0297), and GO:0009744 (Response to sucrose stimulus, *p*-value = 1.46E-05, FDR = 0.0297). The observed down-regulation of such genesets correlates with results of a previous study in *A. thaliana* showing down-regulation of the sugar transporters in response to As stress (Fu et al., 2014). In root up-regulated geneset, regulation of programmed cell death (GO:0043067, *p*-value = 1.00E-05, FDR = 9.47E-03) was also represented as the enriched GO category. Proteomic characterization of the As stress induced roots revealed the accumulation of the lipid peroxidation, and *in vivo* H_2_O_2_ contents (Ahsan et al., 2008). The accumulation of these oxidation related metabolites reflects oxidative stress conditions, which might be lined to cell death. In shoots, geneset enrichment revealed a higher number of regulated GO terms in up-regulated genes as compared to root up-regulated genes. Among the GO terms the enriched were mainly associated with carbohydrate stimulus (GO:0009743, *p*-value = 5.85E-13, FDR = 4.58E-09), response to jasmonic acid (JA; GO:0009753, *p*-value = 2.29E-08, FDR = 1.28E-05), and oxylipin metabolic processes (GO:0031407, *p*-value = 2.78E-05, FDR = 3.63E-03). Previous studies on As stress using proteomics based characterization revealed the up-regulation of the JA pathway and is supported by several additional studies, which recently indicated the role of JA in response to the metal stress (Agrawal et al., 2003; Rodriguez-Serrano et al., 2006; Srivastava et al., 2009).

### Regulatory Gene Categories Responsive to As Stress

**FIGURE 3:**
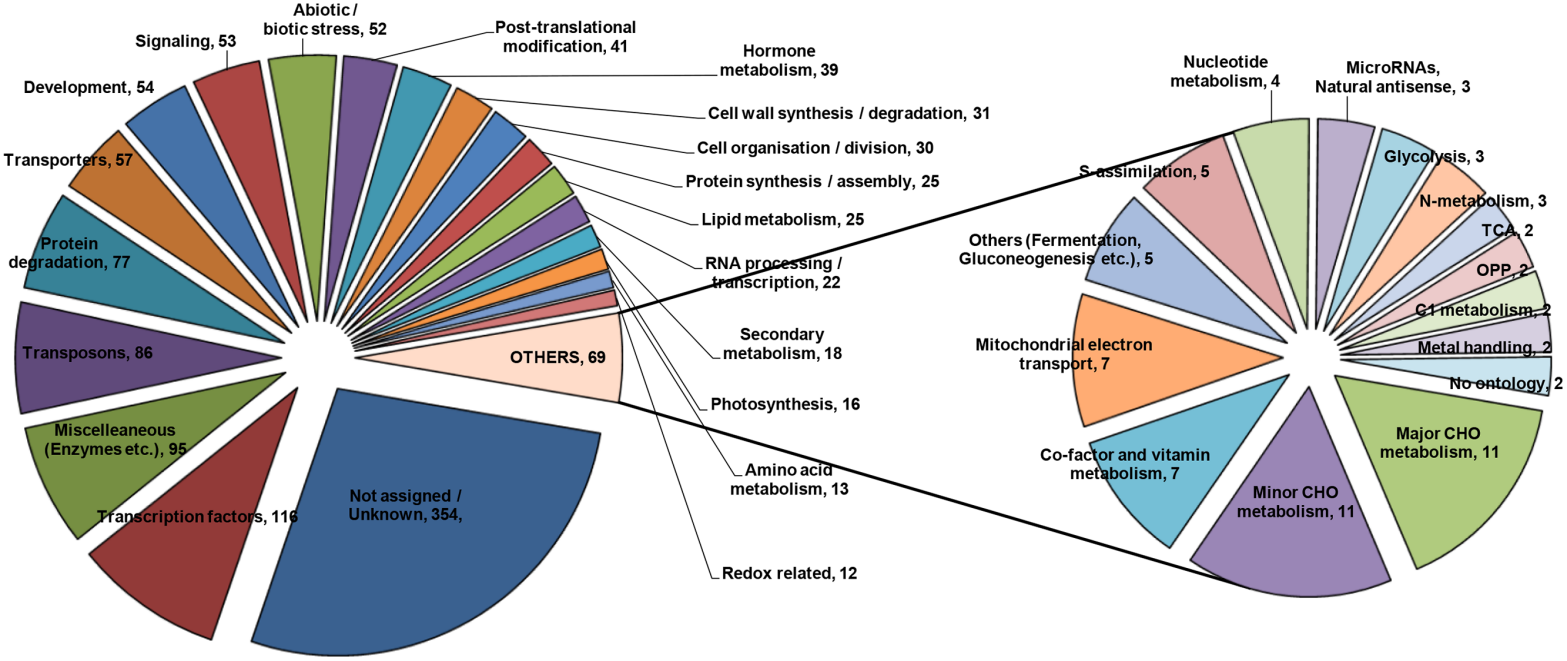
**Metabolic pathway analysis of the DE genes based on KEGG database using SuperViewer online tool**.

To construct a picture from the transcriptome analysis, it was considered imperative to arrange genes into specific functional categories. SuperViewer software^3^ was used for the purpose and genes were classified on the basis of Mapman data. Genes belonging to various major categories were plotted, which is presented in **Figure 3**. A few of the important categories are being discussed in the following sections.

### Transporters: Role of NIPs, PIPs, and Mitochondrial Transporters

**FIGURE 4:**
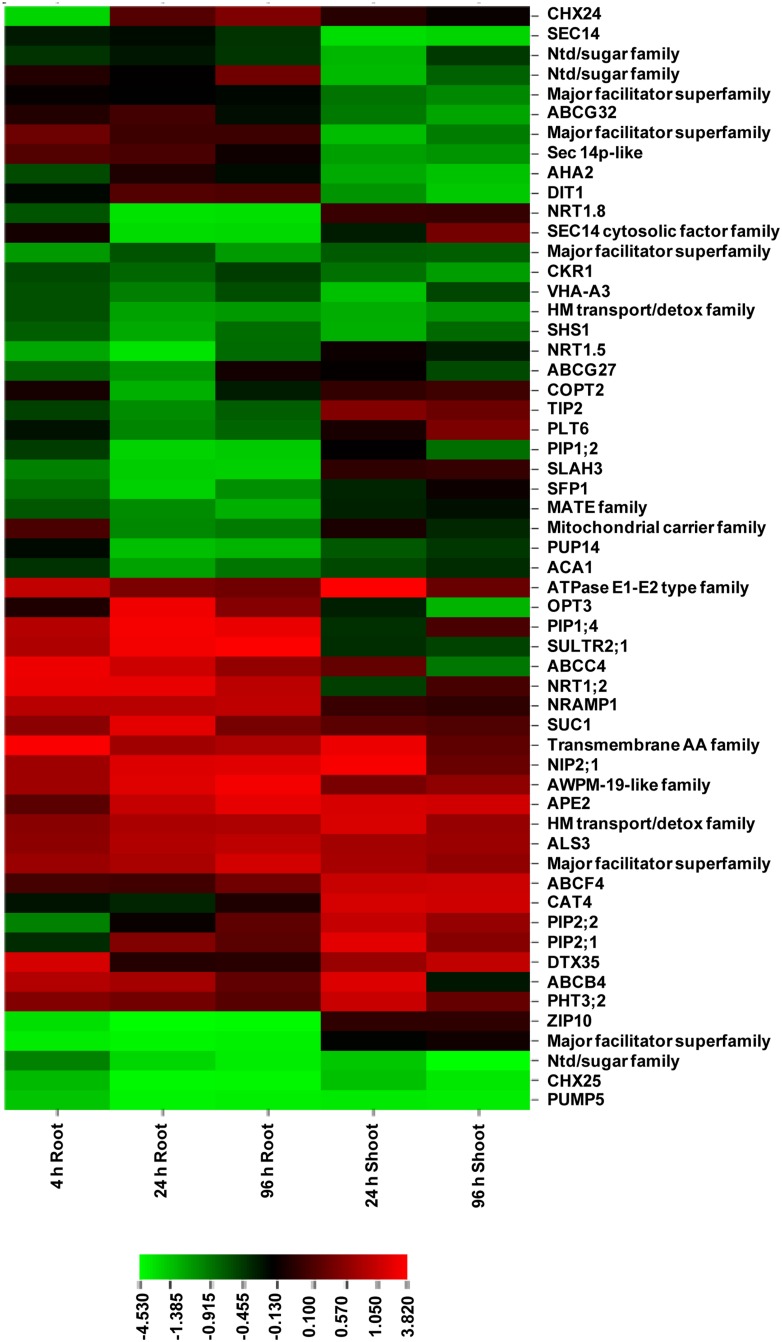
**Expression profile of DE genes encoding transporters at various time points in root and shoot of arsenic-exposed *B. juncea* seedlings.** Red and green represent up-regulated and down-regulated genes, respectively.

Transporters play a key role in the regulation of uptake and transport of As and other metabolites. Superviewer analysis revealed a total of 57 up- and down-regulated transporters in As stress (Supplementary **Data Sheet S4**; **Figure 4**). Among these, we identified six transporters of major intrinsic proteins (MIP) superfamily comprising one nodulin 26-like intrinsic protein (NIP2;1), one tonoplast intrinsic protein (TIP2), and four plasma membrane intrinsic proteins (PIP1;2, PIP1;4, PIP2;1, and PIP2;2). The role of NIP2;1 in rice (OsNIP2;1, known as Lsi1; Low Silicon) in the uptake of As(III) and methylated As species has been previously demonstrated (Ma et al., 2008; Li et al., 2009). In *Arabidopsis* also, role of various NIPs viz., NIP1;1, NIP3;1, and NIP7;1 in AsIII transport has been experimentally demonstrated (Isayenkov and Maathuis, 2008; Kamiya et al., 2009; Xu et al., 2015). As responsiveness of PIPs has been earlier reported by Srivastava et al. (2013b) who suggested that they might regulate water uptake and transport under As stress. Mosa et al. (2012) also proposed a role of certain PIPs (OsPIP2;4, PIP2;6, PIP2;7) in As uptake. Additionally, five ATP-Binding Cassette (ABC) transporters were also found including ABCB4, ABCC4, ABCF4, ABCG27, and ABCG32. ABC transporters were found to be responsive to As(III) in rice also (Yu et al., 2012). Further, five members of mitochondrial transporters were found, which included one phosphate transporter (PHT3;2: up-regulated in shoot), dicarboxylate transporter 1 (DIT1; down-regulated in shoot) and dicarboxylate carrier 1 (DIC1; down-regulated in both shoot and root). Since, As(V) can act as a co-substrate for DIC1 (Palmieri et al., 2008), its down-regulation might be to prevent As(V) entry into mitochondrial matrix so as to avoid disturbance to energy and redox metabolism (Srivastava et al., 2013c).

### Phytohormones: Early Indicators of As Stress

Among the hormone pathways, we specifically focused on JA and ABA pathway owing to their importance as regulators of abiotic stresses (Supplementary **Data Sheet S4**; Supplementary **Figure S7**). Jasmonates are important regulators of As stress perception and response mechanisms (Srivastava et al., 2009). In the present work, genes related to jasmonate biosynthesis and function included allene oxide cyclase 4 (AOC4, down-regulated in root), 12-oxophytodienoate reductases (OPR1, OPR2, and OPR3, up-regulated in both root and shoot), jasmonate resistance 1 (JAR1, down-regulated in shoot), and two jasmonate-zim-domain proteins (JAZ1 and JAZ5, down-regulated in roots). AOC4 and OPRs constitute enzymes of jasmonate biosynthesis pathways, while JAR1 is involved in synthesis of bioactive JA-Isoleucine from JA (Wasternack, 2007; Browse, 2009). JAZs are involved in repression of JA signaling and their degradation leads to jasmonate-dependent gene expression (Staswick, 2008; Ellinger and Kubigsteltig, 2010). Another important gene, sulfotransferase 2 A, which encodes a hydroxyjasmonate sulfotransferase and shows induction upon treatment with methyljasmonate and 12-hydroxyjasmonate, was significantly up-regulated. This gene is supposed to regulate excess JA or biological activity of 12-hydroxyjasmonic acid (Gidda et al., 2003). Among ABA related genes, three important ones were highly ABA-induced PP2C1 (HAI1), ABA insensitive 1 (ABI1), and ABA interacting protein 2 (AIP2). With respect to auxins, genes mostly included those of auxin responsive proteins from shoots and roots, which were up-regulated. In addition, there were two auxin eﬄux carrier family proteins (PIN3 and PIN6), both of which were down-regulated in shoot. Down-regulation of PIN3 and PIN6 appears to be related to inhibition of root growth (Friml et al., 2002; Cazzonelli et al., 2013) under As stress. In addition, SA biosynthesis was also altered as there was consistent up-regulation of an important gene, farnesoic acid carboxyl-*O*-methyltransferase (FAMT) in both roots and shoot. Therefore, various hormones appeared to play important functions in response to As stress in plants with JA and SA being probably the major facilitators as indicated by our pathway enrichment analysis. This is in contrast to earlier study of Yu et al. (2012) where JA was considered to be the major player in signaling of As stress.

### Transcription Factors: Role and Regulation in As Stress

A total of 116 transcription factors showed change in expression upon As stress. These included Myb, WRKY, GATA, AP2/EREBP, heat shock, G2-like, basis helix-loop-helix, homeobox, C2H2 zinc fingers, constants-like, DOF zinc finger etc (Supplementary **Data Sheet S4**; Supplementary **Figures S8A,B**). Two heat shock TFs, HSFA2 and HSFB2A showed significant up-regulation in roots on all studied time points, while HSFA2 also demonstrated significant up-regulation in shoot. HSFA2 is known to act as a key regulator in inducing the defense system under a number of environmental stress as well as against H_2_O_2_ treatment (Nishizawa et al., 2006). One transcription factor, plant U-box 23 (PUB23) was down-regulated in both root and shoot on all time points. PUB23 plays a role in drought stress and negatively regulates water stress response (Cho et al., 2008). Hence, down-regulation of PUB23 might be achieved to balance As-mediated disturbance to water status (Srivastava et al., 2013b). Two important C2H2 zinc finger TFs included zinc finger of *A. thaliana*, ZAT6 and ZAT12, which were significantly down-regulated in root and shoot, respectively. ZAT6 is a repressor of primary root length and regulator of phosphate homeostasis (Devaiah et al., 2007a). ZAT12 forms oxidative stress signal transduction network along with ZAT7 and WRKY25 for the expression of ascorbate peroxidase 1 (APX1; Rizhsky et al., 2004) and plays a central role in ROS homeostasis (Davletova et al., 2005). WRKY family comprised six TFs including WRKY6, WRKY18, WRKY33, WRKY40, WRKY48, and WRKY75. Of these, WRKY75 showed significant up-regulation at 4 h in roots and WRKY6 was up-regulated significantly at 4 h in roots and at 24 h in shoot. In recent analysis of Castrillo et al. (2013) As(V) stress was found to induce burst of transposons and to repress As(V)/Pi transporter PHT1;1 and these responses were mediated by WRKY6. Further, WRKY75 is also known as a modulator of Pi starvation response and root development (Devaiah et al., 2007b). An early induction of WRKY6 and WRKY75 at 4 h thus confirms to the earlier reports that As(V) acts as a Pi mimic and affects expression of genes induced by Pi starvation (Catarecha et al., 2007). Myb family included a total of 13 transcriptional factors showing up or down-regulation at various time points in either root or shoot. Three TFs, Myb15, Myb39, and Myb77 were down-regulated on all time points in both root and shoot. Two other Mybs, Myb28, and Myb73 were both down-regulated in shoots only and have functions in the regulation of glucosinolate metabolism (Augustine et al., 2013) and SA and JA-signaling pathways, respectively (Jia et al., 2011). Another important TF was oxidation-related zinc finger 1 (OZF1; up-regulated in shoot only). OZF1 transcripts get induced in response to H_2_O_2_ and ABA treatments and OZF1 overexpressing plants are relatively resistant to oxidative stress than wild type plants (Huang et al., 2011).

### Signaling Kinetics in As Tolerance

A total of 53 genes were grouped into signaling-related. Signaling related genes included a number of kinases including mitogen-activated protein kinases, calcium and light-mediated genes (Supplementary **Data Sheet S4**; Supplementary **Figure S9A,B**). One leucine-rich repeat receptor like kinase family gene, root hair specific 6 (RHS6) showed an induction of greater than fivefold. This gene is not yet fully characterized; however a study suggested that it might mediate specific external signals for root hair development (Won et al., 2009). Its early induction indicates toward a possible role of RHS6 in perceiving and delivering the As stress signal. A total of 14 genes belonged to calcium signaling category. Calreticulin 3 (CRT3) was found to be up-regulated until 24 h in roots. This is a high affinity Ca^2+^ binding molecular chaperone regulating Ca^2+^ homeostasis in ER lumen (Qiu et al., 2012). Other important known genes included calmodulin-like 38 (CML38; down-regulated in both root and shoot), calcium-dependent protein kinase 4 (CIPK4, up-regulated in both root and shoot), CML42 (up-regulated at 4 h in root and at 24 h in shoot), calcineurin B-like protein 4 (CBL4 or salt overly sensitive 3, SOS3, up-regulated in root and shoot on early time points) and CML1 (up-regulated at 24 h in shoot). CML38 and CML42 play important roles as sensors in Ca^2+^-mediated signaling (Vanderbeld and Snedden, 2007; Vadassery et al., 2013). CML42 acts as a linker connecting Ca(2+) and JA signaling and affects expression of JA responsive genes negatively (Vadassery et al., 2013). CIPK4 is a positive regulator of Ca-mediated ABA signaling through phosphorylation of ABA responsive transcription factors (Zhu et al., 2007). CBL4 along with its interacting kinase CIPK6 (which was also found among responsive genes in this study) modulates the activity of Na^+^ and K^+^ channels to regulate ion homeostasis (Held et al., 2011). Among MAP kinase signaling pathway, MAPKKK3 (down-regulated in shoots), MKK4, and MAPK3 (down-regulated in roots) were altered in response to As. MAPKs functions in a cascade to transducer extracellular signals to the nucleus for cellular adjustments and consists of three components (Sinha et al., 2011). In rice, As(III) stress was found to induce transcripts of MPK3 and MKK4, which were also demonstrated to interact with each other (Rao et al., 2011). In *Brassica*, As stress response appears to involve MAPKKK3–MKK4–MPK3 cascade but its exact nature requires to be analyzed in further studies. FYPP3, a serine threonine protein phosphatase, showed up-regulation of 6.7-fold in 4 h As treated roots and was expressed at control levels on other time points. Such a high level of up-regulation within 4 h of As treatment warrants its important role in As stress perception and signaling, which needs to be evaluated in future work.

### Redox-Related Profiled Genes in As Stress

**FIGURE 5:**
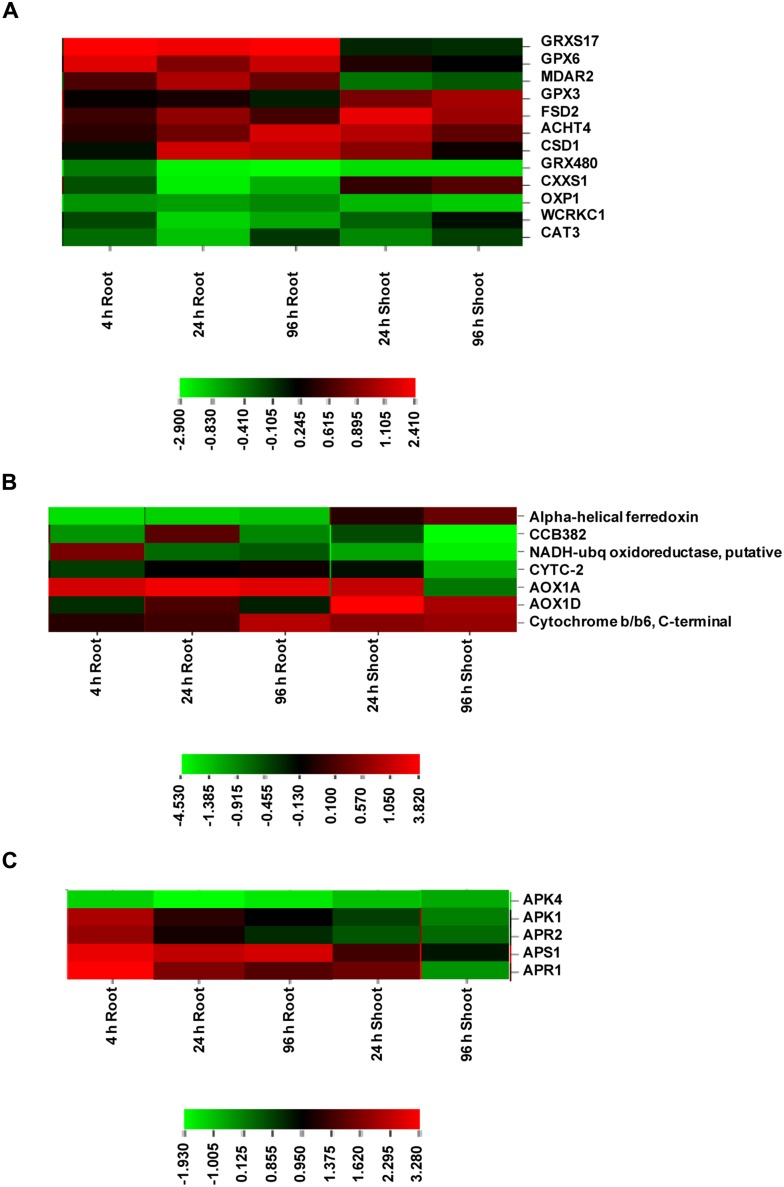
**Expression profile of DE genes encoding proteins of redox (A), electron transport (B), and S-assimilation (C) at various time points in root and shoot of arsenic-exposed *B. juncea* seedlings.** Red and green represent up-regulated and down-regulated genes, respectively.

Redox related genes included a total of 12 genes, of which 7 were up-regulated and 5 were down-regulated (Supplementary **Data Sheet S4**; **Figure 5A**). The up-regulated genes included monothiol glutaredoxin 17 (GRXS17), glutathione peroxidase 6 (GPX6), monodehydroascorbate reductase (MDAR2), copper/zinc superoxide dismutase 1 (CSD1) in roots and GPX3 and Fe SOD 2 (FSD2) in shoot. Glutaredoxins (Grxs) are small ubiquitous proteins of the thioredoxin (Trx) family and mediate reversible reduction of disulphide bonds of their substrate proteins in the presence of GSH via a dithiol or monothiol mechanism (Rouhier et al., 2008). GPX6 encodes cytosolic and mitochondrial isoforms and was found to be strongly up-regulated under various abiotic stresses including copper (Milla et al., 2003). JA has been found to induce specifically the GPX6 expression. GPX3 was found to show a slow increase under various stresses. This is in corroboration with the present transcriptome results. GPX3 has been found to play a dual role in plants: H_2_O_2_ homeostasis and relaying of H_2_O_2_ signal in guard cells. This signal mediates stomatal regulation in response to ABA (Miao et al., 2006). Thus, GPX6 and GPX3 overexpression at different time points appeared to regulate ROS levels and stomatal opening under As stress. SODs constitute the first line of defense against ROS and dismutate superoxide radicals to H_2_O_2_. MDAR is a component enzyme of ascorbate-glutathione pathway, which is involved in regulating H_2_O_2_. Catalase is another important enzyme for H_2_O_2_ regulation. The up-regulation of SODs and MDAR under As stress suggests stimulation of antioxidant machinery to protect against oxidative damage. CAT3 down-regulation appears to be due to differential regulation of three CAT genes (CAT1, CAT2, and CAT3) under various abiotic stress and developmental stages (Du et al., 2008).

### Mitochondrial Electron Transport

All the genes belonging to mitochondrial electron transport chain showed significant up-regulation in both root and shoot including alternative oxidase (AOX1D and AOX1A), cytochrome b/b6 and cytochrome c-2, indicating that ETC was under stress in presence of As due to altered energy requirements (Supplementary **Data Sheet S4**; **Figure 5B**). A significant up-regulation of AOX1 isoforms in this study suggests their critical involvement in the regulation of ROS levels, redox and energy status under As stress (Giraud et al., 2008; Srivastava et al., 2013c). This is in sharp contrast to earlier studies where As treatment failed to induce AOX transcripts during microarray studies in *Arabidopsis* and rice (Abercrombie et al., 2008; Norton et al., 2008; Chakrabarty et al., 2009).

### Transposons

Recent study of Castrillo et al. (2013) elucidated the role of the transposon burst in As stress response, with as many as 869 genes up-regulated in *A. thaliana* plants after 1.5 h exposure to As(V) (Castrillo et al., 2013). Transposon activation in response to stress can lead to deleterious effects, such as gene deletion or insertion, chromosome rearrangement, and alterations in gene expression (Ma and Bennetzen, 2006; Ito, 2012). As proposed by Castrillo et al. (2013) change in transposon expression might be linked to epigenetic alterations and be part of survival mechanisms of plants. A large number of transposons were also found among the responsive genes in this work. This included 37 transposons, which were down-regulated and 38 transposons that were up-regulated on various time points in roots and shoot (Supplementary **Data Sheet S4**; Supplementary **Figure S10**). Only two transposons showed both up-regulation and down-regulation in tissue specific manner. A few of the As-responsive transposons identified by Castrillo et al. (2013) were also present in our study; however, they showed both down- and up-regulation. Further, we observed an up-regulation of WRKY6 gene, which was suggested to be a regulator of transposon expression, at 4 h in roots and at 24 h in shoots in the present study. It thus appears more appropriate to denote these transposons as As(V)-responsive rather than As(V)-inducible. Castrillo et al. (2013) also found that As(V) induction of transposons was transient, with the highest expression at 1.5 h after As(V) exposure. Hence, transposons do appear to play some important role in As stress response of plants but their exact functions and its time dependency is not known, which needs to be delineated in future studies using the profiled genes in ours study as a platform.

### Sulfur Metabolism in As Stress and Inhibitor Treatments to Understand its Interconnections

**FIGURE 6:**
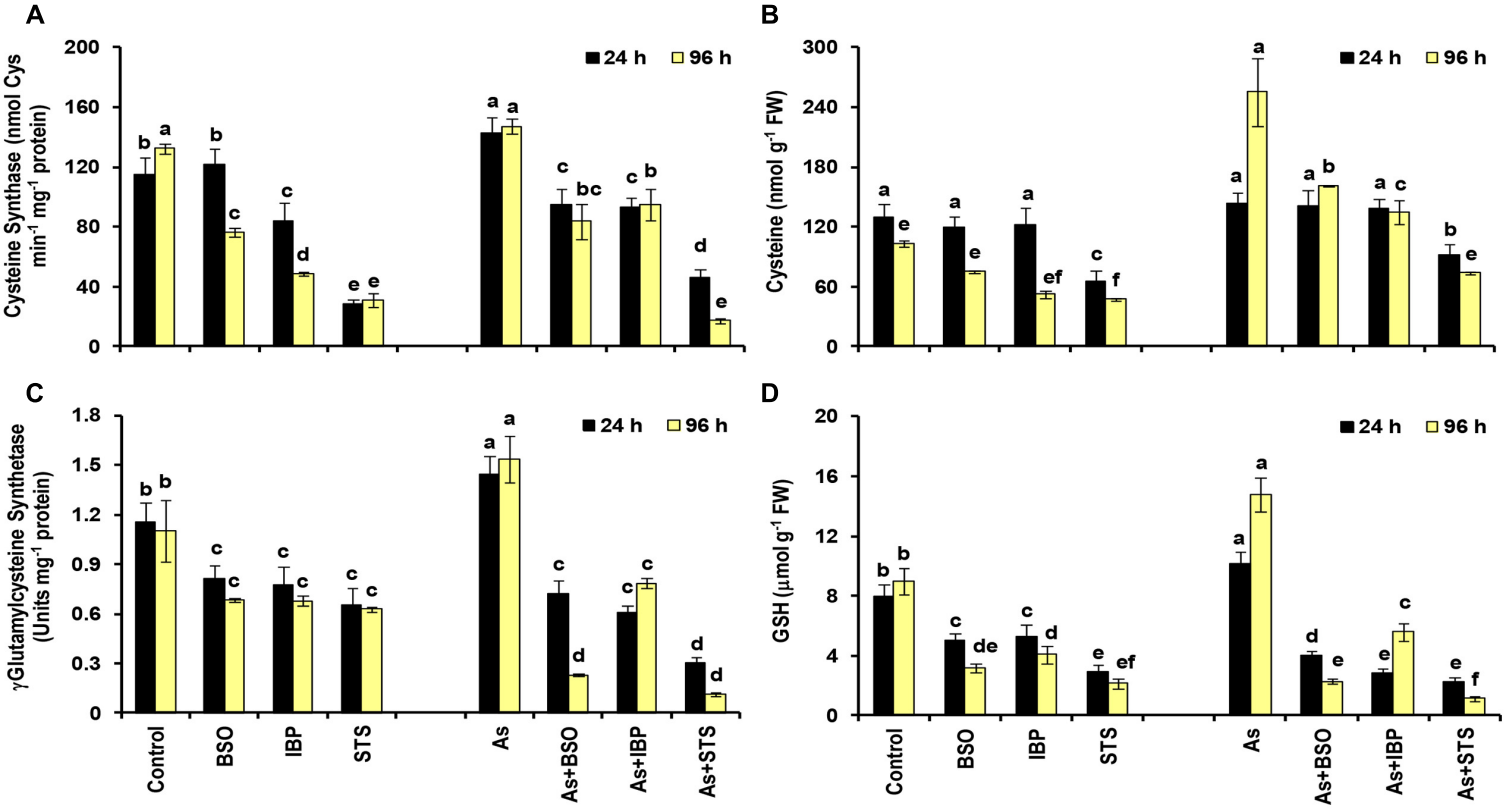
**Effect of Buthionine Sulfoximine (BSO), Ibuprofen (IBP), and Staurosporine (STS) on the activities of cysteine synthase (A) and γ-glutamylcysteine synthetase (C), and on the levels of cysteine (B) and glutathione (D) in control and arsenic-exposed *B. juncea* seedlings at 24 and 96 h.** All values are means of triplicates ± SD. ANOVA significant at *p* ≤ 0.01. Different letters indicate significantly different values at a particular duration (DMRT, *p* ≤ 0.05).

**FIGURE 7:**
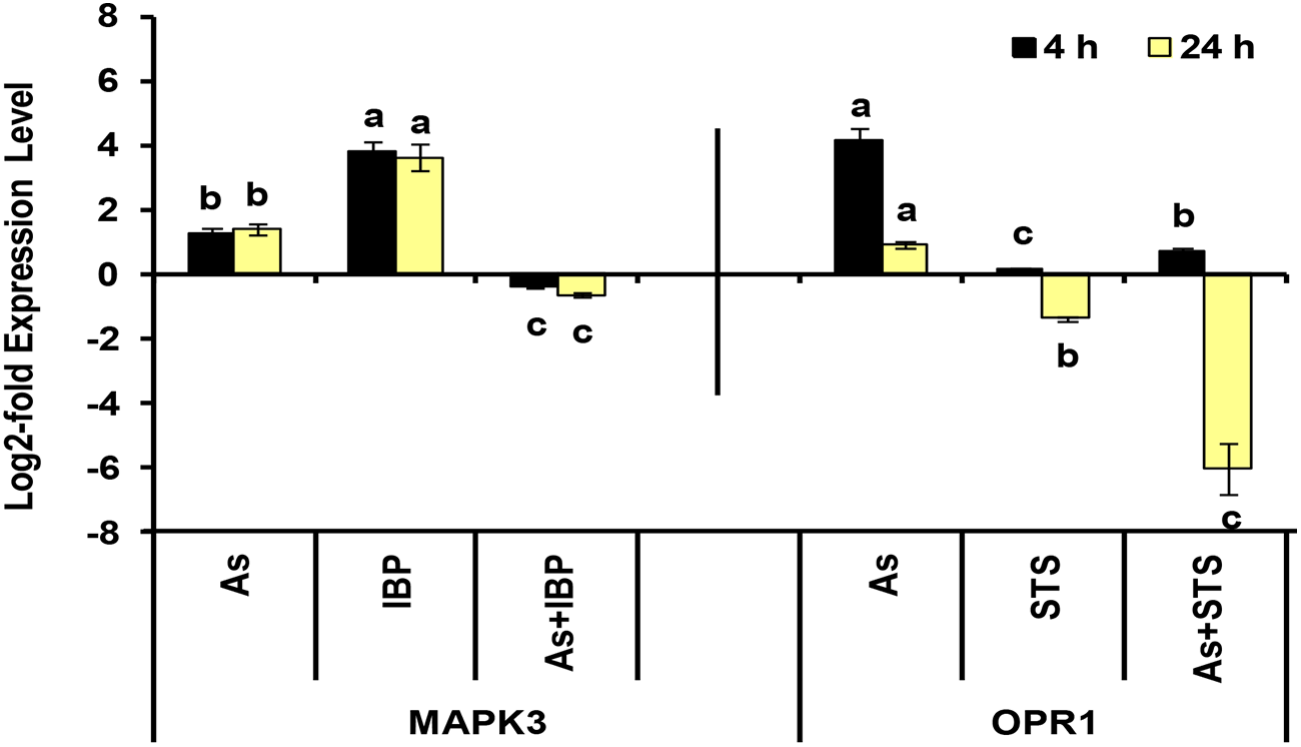
**Effect of IBP and STS on the log2-fold expression level of MAPK3 and OPR1 genes in arsenic-exposed *B. juncea* seedlings at 4 and 24 h.** All values are means of triplicates ± SD.ANOVA significant at *p* ≤ 0.01. Different letters indicate significantly different values at a particular duration for a gene (DMRT, *p* ≤ 0.05).

Among sulfur assimilation pathway, ATP sulfurylase 1 (APS1), adenosine-5′-phosphosulfate reductase (APR1 and APR2) and adenosine-5′-phosphosulfate kinase (APK1) were up-regulated, while APK4 was down-regulated. Further, one sulfate transporter, SULTR2;1 was significantly up-regulated in roots. Hence, sulfur assimilation toward cysteine biosynthesis was more stimulated under As stress, which is in concurrence to earlier biochemical studies (Srivastava et al., 2009; Supplementary **Data Sheet S4**; **Figure 5C**). We further used inhibitors (BSO, IBP, and STS) to understand the interconnection of sulfur metabolism with JA and kinases. The inhibitor treatments were found to have significant impacts on cysteine synthase and γECS activities and on the levels of cysteine and GSH when control and control + inhibitor or As and As + inhibitor treatments were compared (**Figures 6A–D**). Kinase inhibitor produced most significant negative impact on studied parameters. Further, the expression of MAPK3 was affected significantly in presence of jasmonate inhibitor, IBP while the expression of OPR1 was altered in presence of kinase inhibitor STS when As and As+inhibitor treatments were compared (**Figure 7**). Thus, the inhibition of jasmonate or kinase signaling affected the sulfur metabolism. It further affected the expression of important gene of each other. Therefore, sulfur metabolism, jasmonate, and kinase signaling pathways appear to be interconnected and their mutual relationships affects the plants’ responses to As exposure.

## Conclusion

To conclude, we present a genome-wide transcriptional screening to highlight the genes regulated during the As stress and propose candidate genes, which could act as tools for monitoring the early indications of the As stress (**Table 1**). The present study also highlighted the role of the signaling and metabolic pathways in As stress and their dynamic nature with time. We would also like to emphasize that in such a large dataset with several time points and different tissues, there are several genes, which show consistent change that may not, sometimes, be above a threshold, but those genes can certainly carry important functions. Further, there were several unknown genes, which showed significant up-regulation or down-regulation on all time points and in both roots and shoot. Such unknown genes can give new insights into As metabolism in plants.

**Table 1:** List of selected genes, which are functionally important in the context of arsenic response of *Brassica juncea* plants and which can be utilized as early markers of arsenic stress.

Arabidopsis Gene ID	Gene Name	Function	Log2-fold expression (Root)			Log2-fold expression (Shoot)	
			4 h	24 h	96 h	24 h	96 h
At1g32350	AOX1D_alternative oxidase 1D	To maintain metabolic homeostasis during abiotic stress	**1.00**	**1.15**	**1.09**	**2.83**	**1.48**
At3g09350	Fes1A	Cytosolic Hsp70 stability and abiotic stress tolerance	**3.69**	**3.68**	**2.06**	**3.00**	**1.23**
At3g22890	APS1__ATP sulfurylase 1	Sulfur metabolism	**3.03**	**2.29**	**2.39**	**1.37**	0.89
At4g04610	APR1_APS reductase 1	Sulfur metabolism	**3.28**	**1.62**	**1.46**	**1.49**	0.03
At1g76680	OPR1_12-oxophytodienoate reductase 1	Jasmonate biosynthesis	**3.42**	**3.86**	**2.64**	**4.66**	-0.02
At2g26150	HSFA2_heat shock transcription factor A2	Regulator of several environmental stresses	**2.66**	**3.03**	**1.38**	**2.94**	1.65
At4g09570	CPK4_calcium-dependent protein kinase 4	ABA signal transduction	**2.18**	**2.51**	**2.39**	**3.16**	0.31
At5g54160	OMT1_O-methyltransferase 1	Phenylpropanoid metabolism	**4.04**	**3.43**	**1.62**	**2.46**	0.44
At3g09270	GSTU8_glutathione S-transferase TAU 8	Glutathione conjugation	**2.73**	**2.48**	**1.86**	**2.86**	0.27
At1g62300	WRKY6	Regulation of As and phosphate uptake	**1.21**	0.33	0.23	**1.34**	0.47
At4g04950	GRXS17_Monothiol glutaredoxin 17	Involved in ROS regulation and auxin signaling	**2.41**	**2.39**	**2.41**	0.01	-0.02
At5g48850	SDI1_Sulfur-deficiency induced 1	Sulfur deficiency inducible, indicator of sulphur nutritional status	**2.93**	**1.68**	**2.48**	2.01	0.9
At5g59880	ADF3__actin depolymerizing factor 3	Plant abiotic response and tolerance	**3.60**	**1.90**	**2.05**	0.17	0.03
At5g10180	SULTR2;1__slufate transporter 2;1	Sulfur uptake and transport	0.92	**1.73**	**2.3**	-0.38	-0.52
At5g51100	FSD2__Fe superoxide dismutase 2	Superoxide radical dismutation	0.61	0.975	0.615	**1.32**	**1.00**
At2g22500	DIC1_Dicarboxylate carrier 1	Mitochondrial phosphate transport	**-1.38**	**-2.55**	**-2.53**	**-2.29**	**-2.51**

*Values in bold represent significant change in expression.*

## Conflict of Interest Statement

The authors declare that the research was conducted in the absence of any commercial or financial relationships that could be construed as a potential conflict of interest.

## Footnotes

1 https://www.arabidopsis.org

2 http://bbc.botany.utoronto.ca/ntools/cgi-bin/ntools_classification_superviewer.cgi

3 http://bbc.botany.utoronto.ca/ntools/cgi-bin/ntools_classification_superviewer.cgi

## Supplementary material

Supplementary File S1 **Provides information of primers used for Real-time RT PCR analysis (Supplementary Table S1) and contains all supplementary figures (Supplementary Figures S1–S10) of clustering and networking analyses**.

Supplementary Data Sheet S1 **List of *Arabidopsis* gene IDs for Brassica custom IDs**.

Supplementary Data Sheet S2 **List of profile genesets clustered using STEM tool for root and shoot**.

Supplementary Data Sheet S3 **List of co-regulated network genesets analyzed using ReviGo and Gene ontologies and pathway enrichment analyses conducted using PlantGSEA**.

Supplementary Data Sheet S4 **List of all genes showing differential expression in microarray analysis and their categorization in various pathways/functional categories**.
