# Supplementary material for: Transcriptomics profiling of Indian mustard (Brassica juncea) under arsenate stress identifies key candidate genes and regulatory pathways
Source: Front Plant Sci. 2015 Aug 19;6:646. doi: 10.3389/fpls.2015.00646 (PMC4541038; doi:10.3389/fpls.2015.00646)
Supplement: Supplementary File S1 — Provides information of primers used for Real-time RT PCR analysis (Supplementary Table S1) and contains all supplementary figures (Supplementary Figures S1–S10) of clustering and networking analyses. [file File_1.DOCX]

**Supplementary Information**

**Transcriptomics profiling of Indian mustard (*Brassica juncea*) under arsenate stress identifies key candidate genes and regulatory pathways**

Sudhakar Srivastava^1#*$^, Ashish Kumar Srivastava^1$^, Gaurav Sablok^2^, Tejaswini U. Deshpande^3^and Penna Suprasanna^1^

^1^ Nuclear Agriculture and Biotechnology Division, Bhabha Atomic Research Centre, Mumbai-400085, Maharashtra, India; ^2^ Plant Functional Biology and Climate Change Cluster (C3), University of Technology Sydney, PO Box 123, Broadway, NSW 2007, Australia; ^3^Shri J.J.T. University, Vidyanagari, Jhunjhunu-333001, Rajasthan, India

# Current Address: Institute of Environment & Sustainable Development, Banaras Hindu University, Varanasi-221005, U.P., India

$ Authors contributed equally to this work

*Corresponding author:

E-mail: sudhakar.srivastava@gmail.com; sudhakar.iesd@bhu.ac.in

**Supplementary Table 1**: Primer sequences of genes used for real time RT-PCR analysis

| Gene Name | Forward primer | Reverse primer |
| --- | --- | --- |
| Actin | TGGGTTTGCTGGTGACGAT | TGCCTAGGACGACCAACAATACT |
| PIP1;2 | Ccagcggttacgttcggtttgtt | Ccgatagggagcggtgctagaat |
| PIP2;2 | Tgattagggcggtgctttacatgg | Tggaacctgaagcccttaggaca |
| SULTR2;1 | TTTTGAATCTCTCTCACATCAAGTTCTCCC | TGGTCTTGACCGGCTTGTGCG |
| APS1 | AGGCTGGACAAGTCCACTCGG | GCCGTCGTCAAGACGTAGCGA |
| FSD2 | AACCGGCGAATGCAATGGAAAAG | CCTCCACCTCCAGGTTGGATAGACT |
| CAT3 | CATCGCCTTGGACCGAATTATTTGC | TGAGACGTGGCTCCGATAGAATCTC |
| OPR1 | TTGCTGCAAGAAATGCTATGGAAGC | CATCGGCATTAGTGTGTGAGGACA |
| CTR1 | CGTCGCCTGAGTATGGCTTATGATG | TGCAACCTGAGGATTCAGATTACGC |
| DOF5.8 | TCTGCCGCTACCACCTCCGT | AGTTGCAGCATCAGTAGCGCCAC |
| WRKY33 | GTTGGTGACGATGAGTTCGAACAAG | TTCCTCACTGGACAACCGATGG |
| WRKY6 | CCCGTTCGCAAACAAGTTCAA | TGCCTGGCCTATTACATGAGG |
| ACA13 | TGCGATGGCAATGCAAACTCCC | GGACACACTCGGTGCATTGGCTT |
| MAPK3 | GCACACCGACAGAATCTGATCTCG | TGCTGCACTTCTAACCGTATGTTGG |

**Supplementary Figure 1**: Differential genes from Root data were profiled into 20 clusters from which 6 clusters are identified as significantly ordered based on their p-values. Profile color represents the significant cluster and same color profile belongs to the same cluster. Number in top left-hand corner is profile ID and down left-corner is p-value. Here Profile 17 & 19 are from same clusters. Similarly, profile 0 and profile 4 belongs to the same cluster. Significant down regulated genes were clustered into 3 profiles as, Profile 0 (P0) , Profile 4 (P4) and Profilre10 (P10) considered as down regulated profiles. While, up regulated genes clustered into Profile 17(P17), Profile 18 (P18) and Profile 19 (P19) considered as up regulated profiles (based on Ernst et al, BMC Bioinformatics2006 ).Expression pattern of P0 (n=115) ,P4 (n=113) and P10 (n=84) genes illustrates down-regulation of genes and P17 (n=83) ,P18 (n=67) and P19 (n=164) genes illustrates up-regulation of genes at each time point. Respective hierarchical clustering with Pearson’s distance metric showed clustering of temporal Root samples and gene cluster down and up-regulation pattern.

**Supplementary Figure 2**: Differential genes from Shoot data were profiled into 20 clusters from which 5 clusters identified as significant ordered based on their p-values. Profile color represents the significant cluster and same color profile belongs to the same cluster. Number in top left-hand corner is profile ID and down left-corner is p-value. Here Profile 11 & 14 are from same clusters. Similarly, profile 0 and profile 4 belongs to the same cluster. Significant downregulated genes were clustered into 2 profiles as, Profile 0 (P0) and Profile 4 (P4) considered as downregulated profiles. While, upregulated genes clustered into Profile 11(P11), Profile 14 (P14) and Profile 15 (P15) considered as upregulated profiles (JasonErnst et al, BMC Bioinformatics2006 ).Expression pattern of P0 (n=48) ,P4 (n=168) illustrate down-regulated gene profiles and P11 (n=127), P14(n=86) and P15(n=42) illustrates up-regulation of genes at each time point. Respective hierarchical clustering with Pearson’s distance metric showed clustering of temporal Root samples and gene cluster shows down-regulation and up-regulation pattern.

**Supplementary Figure 3**: Pathway analysis of profile (P0,P4 and P10) genes of roots using ReviGo. The profile genes distributed into GO terms as Biological process, Cellular component and Molecular function based on their functions. Pathway node color and larger node size indicates the enrichment of p-value; Dark red specifies the significant p-value. Highly similar GO terms are linked by edges in the graph and the line width indicates the degree of similarity.

**Supplementary Figure 4**: Pathway analysis of profile (P17,P18 and P19) genes of roots using ReviGo. The profile genes distributed into GO terms as Biological process, Cellular component and Molecular function based on their functions. Pathway node color and larger node size indicates the enrichment of p-value; Dark red specifies the significant p-value. Highly similar GO terms are linked by edges in the graph and the line width indicates the degree of similarity.

**Supplementary Figure 5**: Pathway analysis of profile (P0 andP4) genes of shoot using ReviGo. The profile genes distributed into GO terms as Biological process, Cellular component and Molecular function based on their functions. Pathway node color and larger node size indicates the enrichment of p-value; Dark red specifies the significant p-value. Highly similar GO terms are linked by edges in the graph and the line width indicates the degree of similarity.

**Supplementary Figure 6**: Pathway analysis of profile (P11,P14 and P15) genes of shoot using ReviGo. The profile genes distributed into GO terms as Biological process, Cellular component and Molecular function based on their functions. Pathway node color and larger node size indicates the enrichment of p-value; Dark red specifies the significant p-value. Highly similar GO terms are linked by edges in the graph and the line width indicates the degree of similarity.

**Supplementary Figure 7.** Expression proﬁle of differentially expressed genes encoding proteins of phytohormone metabolism and response at various time points in root and shoot of arsenic-exposed *Brassica juncea* seedlings. Red and green represent up-regulated anddown-regulated genes, respectively.

**Supplementary Figure 8 A and B.** Expression proﬁle of differentially expressed genes encoding transcription factors at various time points in root and shoot of arsenic-exposed *Brassica juncea* seedlings. Red and green represent up-regulated anddown-regulated genes, respectively.

**Supplementary Figure 9 A and B.** Expression proﬁle of differentially expressed genes encoding proteins of signaling pathways and post-translational modification at various time points in root and shoot of arsenic-exposed *Brassica juncea* seedlings. Red and green represent up-regulated anddown-regulated genes, respectively.

**Supplementary Figure 10.** Expression proﬁle of differentially expressed transposons at various time points in root and shoot of arsenic-exposed *Brassica juncea* seedlings. Red and green represent up-regulated anddown-regulated genes, respectively.

**Supplementary Figure 7**

**Supplementary Figure 8 A**

**Supplementary Figure 8 B**

**Supplementary Figure 9 A**

**Supplementary Figure 9 B**

**Supplementary Figure 10**
